# Desires and Aversions

**Published:** 1884

**Authors:** Wm. Jefferson Guernsey

**Affiliations:** 4430 Frankford Avenue, Philadelphia


### DESIRES AND AVERSIONS.

In the choice of a remedy, Hahnemann teaches us that it is not only the *prominent* symptoms, or those most complained of, which should be noted, but also the *uncommon** and *peculiar* (characteristic) features of the case, " which," he says, " *in particular should bear the closest similitude to the symptoms of the desired medicine.*" Not that a new medicine should be administered for every " *peculiar* " symptom that may arise, but that these oddities should be considered in the selection of the remedy. General symptoms are " common to every disease and almost every drug." The *un*common sensations and pains, likes and dislikes, are simply the " guiding symptoms " to the choice of the remedy that will often be found to cover the " totality of symptoms " in the case.

WM. JEFFERSON GUERNSEY, M. D.,
4430 Frankford Avenue, Philadelphia.

December 7th, 1883.

### DESIRE FOR

1. ACIDS:

| | | |
|---|---|---|
| Adelhe. | Cistus. | Phella. |
| Alumin. | Conium. | *Phosph.* |
| Am. car. | Corn. c. | Plumbm. |
| Am. mur. | Cor. ru. | *Podoph.* |
| *Ant. cr.* | Cubeba. | Poly. o. |
| *Ant. ta.* | Cu. ace. | *Pul. pr.* |
| Arg. ni. | *Digita.* | *Sabadl.* |
| *Arnica.* | Elap. c. | Sabina. |
| *Ars. al.* | Fer. me. | Saccha. |
| Arundo. | Granat. | *Secale.* |
| Bellad. | *Hep. sl.* | *Sepia.* |
| Borax. | Hippom. | *Squill.* |
| Bromin. | *Ignati.* | *Stramo.* |
| *Bryoni.* | Ka. bic. | *Sulphu.* |
| Cal. cb. | *Ka. car.* | Thea. c. |
| Carb. a. | Kreoso. | Therid. |
| Carb. v. | *Laches.* | Thuj. o. |
| *Chamom.* | Mag. cb. | Ustila. |
| Che. ma. | Mangan. | VER. AL. |
| Chi. of. | Me. j. f. | Zizia. |

2. " CITRIC: *Pul. pr.* Ver. al.

* See *Organon*, ¶ 153.

### DESIRE FOR

3. ACID DRINKS:

| | | |
|---|---|---|
| Am. mur. | Corn. c. | Plumbm. |
| Ant. cr. | Digita. | Secale. |
| Borax. | Hep. sl. | Squill. |
| Bryoni. | Ka. bic. | Stramo. |
| Cal. cb. | Me. j. f. | Ver. al. |
| Chamom. | Phella. | |

4. " FRUITS: Adelhe. Ars. al. Cal. cb. Chi. of. Ignati. Thuj. o.

— " LEMONS=68.

— PICKLES=90.

— " VINEGAR=125.

5. ALCOHOLIC DRINKS. (Compare Brandy, 15, Claret, 27, Whiskey, 131, and Wine, 132.)

| | | |
|---|---|---|
| Alcohol. | Cu. met. | Naja. t. |
| Aloe. s. | (Ginsen.) | *Pul. pr.* |
| Am. car. | Hep. sl. | So. t. a. |
| Ant. ta. | Iodium. | Staphs. |
| *Arnica.* | Laches. | Sulphu. |
| Ars. al. | LAC. CN. | Sul. ac. |
| Ast. ru. | Na. pho. | Tabacm. |

6. ALE (compare Beer 11): Fer. ph.

— ALL THINGS=45.

7. ALMONDS: Cubeba.

8. APPLES: Aloe. s. *Ant. ta.*

9. APPLE-BUTTER: Hydrph.

10. BACON: Mezeri.

11. BEER. (Compare Ale, 6.)

| | | |
|---|---|---|
| *Acon. n.* | (Coc. ca.) | *Phella.* |
| (Agar. m.) | Colocy. | (Phosph.) |
| Aloe. s. | Graphi. | *Phos. ac.* |
| Am. car. | Ka. bic. | Psorin. |
| (Ant. cr.) | *Laches.* | *Pul. pr.* |
| Ant. ta. | Mangan. | *Rhs. tx.* |
| Asarum. | Me. sol. | *Sabadl.* |
| *Bryoni.* | *Me. viv.* | *Spigel.* |
| Cal. cb. | Moschs. | Spongi. |
| Caladi. | *Na. car.* | Stramo. |
| Camphr. | Na. mur. | *Stront.* |
| Carb. s. | Na. pho. | *Sulphu.* |
| *Caustc.* | NUX VM. | Tellur. |
| Chi. of. | Opium. | Zn. met. |
| *Coccul.* | *Petrol.* | |

12. BISCUIT: Plumbm.

13. BITTER (bitter things in general):

| | | |
|---|---|---|
| Codein. | *Digita.* | Na. Mur. |

### DESIRE FOR

14. BITTER DRINKS : Na. mur.
— BLACK COFFEE=32.
— BOILED EGGS : (soft)=44.
15. BRANDY :

| | | |
|---|---|---|
| Acon. n. | Coca. | Pul. pr. |
| (Ailant.) | Cubeba. | *Seleni.* |
| Arg. ni. | Fer. ph. | *Sepia.* |
| *Ars. al.* | *Hep. sl.* | *Spigel.* |
| Bovist. | Laches. | *Staphs.* |
| Bryoni. | Moschs. | *Sulphu.* |
| Cal. cb. | Mur. ac. | *Sul. ac.* |
| Chi. of. | *Nux vm.* | Therid. |
| Cicu. v. | OPIUM. | |

16. BREAD :

| | | |
|---|---|---|
| Aloe. s. | ·Fer. mt. | Opium. |
| Am. car. | Gratio. | *Plumbm.* |
| *Ars. al.* | Hel. ni. | Pul. pr. |
| Au. met. | Hydras. | (Secale.) |
| Bellad. | Ignati. | Silicea. |
| Bovist. | Mag. cb. | Staphs. |
| Can. in. | Na. car. | *Stront.* |
| (Cast. e.) | *Na. Mur.* | Sumbul. |
| Colocy. | Oleum. | |

17. " BUTTERED :

| | | |
|---|---|---|
| Agar. m. | Ignati. | Me. viv. |
| Bellad. | *Mag. cb.* | (Pul. pr.) |
| *Fer. mt.* | ME. SOL. | |

18. " RYE : Carlsb.
19. " WHEAT : *Au. met.*
— BURNED COFFEE=33.
20. BUTTER : Me. viv.
— " APPLE=9.
— " on BREAD=17.
21. CAKES : Plumbm.
— CAYENNE PEPPER=89.
— CHALK=70.
22. CHARCOAL : Alumin. *Cicu. v.* Conium. Nit. ac. Nux vm.
23. CHEESE : Ast. ru. Ignati.
24. CHERRIES : Chi. of.
25. CHOCOLATE : Hydroph. Lepidi.
26. CIDER : Benz'n.
— CITRIC ACID=2.
27. CLARET : Cal. sl.
— CLAY=70.
28. CLOVES : Alumin.
29. COAL : Cicu. v.

### DESIRE FOR

30. COARSE FOOD: Ab. can.

31. COFFEE:

| | | |
|---|---|---|
| ANGUST. | Capsic. | Moschs. |
| Arg. mt. | Carb. v. | Na. mur. |
| Arg. ni. | Chamom. | Nux ms. |
| *Ars. al.* | *Chi. of.* | Nux vm. |
| Ast. ru. | Colchi. | Pho. ac. |
| *Au. met.* | *Conium.* | *Selini.* |
| *Bryoni.* | Granat. | So. t. a. |
| Cal. ph. | Lepidi. | Sulphu. |

32. " BLACK: Moschs.

33. " BURNED: Alumin.

34. COLD DRINKS:

| | | |
|---|---|---|
| Am. car. | Colchi. | *Na. slf.* |
| *Angust.* | Croc. s. | *Oleand.* |
| Ant. ta. | Digita. | Phosph. |
| Ars. al. | Dulcam. | Pho. ac. |
| Au. met. | (Eu. per.) | Plumbm. |
| *Bovist.* | Euphor. | Psorin. |
| Bryoni. | Ka. bic. | Rhs. tx. |
| Cal. cb. | Ka. nit. | Ruta g. |
| Caustc. | Ledm. p. | *Sabadl.* |
| Chamom. | Mag. cb. | Spigel |
| Chi. of. | Me. cor. | Sulphu. |
| *Cina.* | Me. sol. | Thuj. o. |
| Clemat. | *Me. viv.* | *Ver. al.* |
| Coccul. | Na. mur. | Vipera. |

35. " FOOD:

| | | |
|---|---|---|
| (Am. car.) | Cu. met. | Silice. |
| (Ant. ta.) | Me. sol. | Thuj. o. |
| Cu. ars. | (Na. mur.) | *Ver. al.* |

— " MILK=77.

— " WATER=129.

36. COOKED FOOD: Nit. ac.

— CONSTANT, TO EAT=41.

— CRESS=130.

37. CUCUMBERS: *Ant. cr.* Ver. al.

— DAINTIES=38.

38. DELICACIES:

| | | |
|---|---|---|
| Bufo c. | Cu. met. | Rhs. tx. |
| Cal. cb. | Ipecac. | Sangui. |
| Chi. o. | Petrol. | Spongi. |

— DRINKS, ACID=3.

— " ALCOHOLIC=5.

— " BITTER=14.

— " COLD=34.

### DESIRE FOR

38. DRINKS, HOT=60.
— " SOUR=3.
— " WARM=126.
39. " WITHOUT THIRST: Camphr. Colocy. Wiesba.
— DRY RICE=101.
— EARTH=70.
40. EAT, CANNOT, WHAT HE—: Bryoni.
41. " CONSTANT:

| | | |
|---|---|---|
| Bovist. | Myrica. | Ratanh. |
| (Granat.) | Na. car. | Tabacm. |
| Me. viv. | | |

42. EGGS: Hydras. Na. pho.
43. " FRIED: Na. pho.
44. " SOFT BOILED: Cal. cb. Ol. ani.
45. EVERYTHING: Santon.
46. FARINACEOUS FOOD: Sabadl. Sumbul.
47. FAT FOOD. (Compare Ham fat, 56.) *Nit. ac.* *Nux vm.*
— " HAM=56.
48. FISH: (Na. mur.)
— " HERRING=58.
— " SARDINES=105.
49. FLOUR: Cal. cb. Sabadl.
— " DISHES OF=73.
50. " UNCOOKED. Cal. cb.
— FOOD, ACID=1.
— " BITTER=13.
— " COLD=35.
— " COOKED=36.
— " FARINACEOUS=46.
— " FAT=47.
— " FRIED=53.
— " HEARTY=57.
— " INDIGESTIBLE=63.
51. " INSATIABLE:

| | | |
|---|---|---|
| (Asc. tu.) | Au. met. | *Spongi.* |
| Ant. cr. | Bar. cb. | Squill. |
| Arg. mt. | Fer. mt. | Stannm. |
| (Arg. ni.) | (Petrol.) | Staphs. |
| Angust. | Pul. nu. | *Zn. met.* |
| Arum t. | *Secale.* | |

— " LIQUID=71. (Also SOUPS, 111.)
52. " SATIETY, SUDDEN, ON EATING:

| | | |
|---|---|---|
| Agar. m. | *Bar. cb.* | Coc. ca. |
| Am. car. | Bryoni. | Colchi. |
| Ant. ta. | Cicu. v. | Conium. |
| Ars. al. | Coca. | Croc. s. |

### DESIRE FOR

52. FOOD, SATIETY, SUDDEN, ON EATING.

| | | |
|---|---|---|
| Cyclam. | Mag. cb. | Rheum. |
| Gelsem. | Me. sol. | *Rhodod.* |
| Guarea. | Me. viv. | Serpen. |
| Hyperi. | *Na. mur.* | Spongi. |
| Ignati. | Nux ms. | Thea c. |
| Ledm. p. | Prun. s. | Thuj. o. |
| LYCOPO. | | |

— " SMOKED=108.
— " SOFT=110.
— " WARM=127.
53. FRIED FOOD: Plumbm.
— " EGGS=43.
54. FRUIT:

| | | |
|---|---|---|
| Aloe s. | Cubeba. | Pho. ac. |
| Alumen. | Granat. | Pul. pr. |
| *Alumin.* | *Ignati.* | *Sul. ac.* |
| *Ant. ta.* | Mag. cb. | VER. AL. |
| Chi. of. | | |

— " ACID=4.
— " APPLES=8.
— " CHERRIES=24.
55. " GREEN: Cal. cb. Lepita.
— " LEMONS=68.
— " ORANGES=85.
— " PLUMS=92.
— " GREEN FRUIT=55.
56. HAM FAT: Mezeri.
57. HEARTY FOOD: Rhs. tx. Ustila.
58. HERRING: *Nit. ac.* *Ver. al.*
59. HONEY: Sabadl.
60. HOT DRINKS: Cas. car. Che. ma.
61. ICE: Elap. c. Me. cor.
62. ICE-CREAM: Eu. per.
— IMPOSSIBLE THINGS=123.
— INDEFINITE THINGS=64.
63. INDIGESTIBLE THINGS: Alumin. Bryoni.
64. INDISTINCT:

| | | |
|---|---|---|
| Bryoni. | Laches. | Sangui. |
| *Chi. of.* | *Mag. mu.* | Silice. |
| Ignati. | *Pul. pr.* | *Therid.* |
| Ipecac. | | |

— INSATIABLE FOR FOOD=51.
65. JUICES: Aloe s. Granat. Na. ars. *Pho. ac.*
66. JUICY FRUITS: Sarsap.
67. LARD: Ars. al.

### DESIRE FOR

68. LEMONS: *Ars. al.* Benzoi. Nabalu.

69. LEMONADE. (Compare ACID DRINKS, 3.)

Bellad. *Sabadl.* Sabina. Secale.

70. LIME, SLATE PENCILS, EARTH, CHALK, OR CLAY.

Alumin. Cicu-v. *Nit. ac.* *Nux vm.*

71. LIQUID FOODS. (Compare SOUPS, 111, warm liquid food, 128.)

*Angus.* Bryoni. *Fer. mt.* Me. viv. *Staphs.* *Sulphu.* Ver. al.

72. " FOODS, WITHOUT THIRST: Bellad.

— MALT LIQUORS=ALE, 6, or BEER, 11.

— MANY=82.

73. MEAL, DISHES OF: Laches. *Sabadl.*

74. MEAT:

Ab. can. Aloe s. Au. met. Fer. mt. (Graphi.) Hel. ni. *Lil. ti.* *Mag. cb.* *Mengan.* Me. sol. Me. viv. Na. mur. Sabadl. Sulphu.

— " BACON=10.

— " SALT=104.

75. " SMOKED: Caustic.

76. MILK:

Anacar. *Ars. al.* *Au. met.* Baptis. (Borax.) Bovist. *Bryoni.* *Cal. cb.* *Che. ma.* Elap. c. Ka. jod. Mag. cb. (Mangan.) Me. sol. *Me. viv.* Na. mur. *Nux vm.* Phellan. *Pho. ac.* Rhs. tx. *Sabadl.* Sabina. *Silice.* *Staphs.* *Stront.* Sulphu.

77. " COLD: Phellan. Phor. ac. Rhs. tx. Sabadl. Staphs.

78. " SOUR: Ant. ta.

79. NARCOTICS: Tabacm.

80. NIBBLE: (Bar. cb.) Mag. mu. Na. car.

81. NOTHING: Ars. al. Coccul. Hep. sl. Linum. Mezeri. Millef. Niccol.

82. NUMEROUS: CINA. Phosph.

83. NUTS: Cubeba.

— " ALMONDS=7.

84. ONIONS: Cubeba.

— OPIATES=79.

85. ORANGES: Cubeba. Elap. c. So. t. a.

### DESIRE FOR

86. OYSTERS: Apis. m. Bryoni. *Laches.* Lycopo. Rhs. tx.
87. " RAW: Rhs. tx.
88. PASTRY: Bufo c. Plumbm.
89. PEPPER, CAYENNE: Me. cor.
90. PICKLES: Ab. can. Hep. sl. (Hyperi.) (Na. ars.) Staphs. Ver. al.
91. PIQUANT THINGS. (Compare SEASONED THINGS, 107, SPICED FOOD, 113, STRONG FOOD, 115.) Flu. ac.
92. PLUMS=Sul. ac.
93. POISON, ANOTHER DRAUGHT OF: Opium.
— PORK, BACON=10.
94. POTATOES: Ol. ani.
95. " RAW: Cal. cb.
96. PUDDINGS: Sabadl.
— PUNGENT FOOD=91.
97. RAGS, CLEAN: Alumin.
98. RAW FOOD: Silice. Tarent.
— " OYSTERS=87.
— " POTATOES=95.
— RED PEPPER=89.
99. REFRESHING THINGS:

| | | |
|---|---|---|
| Carb. a. | *Pho. ac.* | Sangin. |
| *Caustc.* | *Pul. pr.* | Tilia. |
| *Coccul.* | Rheum. | Ver. al. |
| *Phosph.* | Sabina. | *Valeri.* |

100. RICE: Terebi.
101. " DRY: Alumin.
—— RICH FOOD, PASTRY=88, FAT, 47, PUDDINGS, 96.
—— RYE-BREAD=18.
102. SALAD: Elap. c. Lepita.
103. SALTS: (Salt things.)

| | | |
|---|---|---|
| Atropi. | Cor. ru. | *Nit. ac.* |
| *Cal. cb.* | Mephit. | Phosph. |
| Carb. v. | Me. j. f. | Seleni. |
| *Caustc.* | Me. j. r. | Thuj. o. |
| *Conium.* | Na. mur. | *Ver. al.* |

104. SALT MEAT: (Arg. ni.) Cori. r.
105. SARDINES: *Ver. al.*
106. SAUCE, with food: Nux vm.
107. SEASONED FOOD: Flu. ac. Hep. sl.
—— SLATE PENCILS=70.
—— SMOKE=122.
108. SMOKED THINGS: *Caustc.*
—— " MEAT=75. Bacon=10.
109. SNOW: Crot. c.
—— SOFT-BOILED EGGS=44.

### DESIRE FOR

110. Soft food: Alumen. Pyrus. Sulphu.
111. Soups. (Compare liquid foods, 71, warm liquid food, 128.) Bryoni. Na. mur. Phellan.
—— Sours. (Sour things in general)=1.
112. Sour-krout: *Carb. a.* Cham.
—— " milk=78.
113. Spiced food. (Compare Piquant, 91, seasoned, 107, strong food, 115.)
Ast. ru. *Hep. sl.* Phosph. *Sangin.*
—— Spirituous liquors=5.
114. Starch: Alumin. Nit. ac.
115. Strong food. (Compare Piquant, 91, seasoned, 107, spiced, 113). Na. pho.
116. Sugared water. (Compare sweets, 117). Bufo. c. Sulphu.
117. Sweets:

| | | |
|---|---|---|
| *Am. car.* | *Ka. car.* | Petrol. |
| Arg. mt. | *Lycopo.* | Plumbm. |
| Bar. cb. | *Mag. mu.* | *Rheum.* |
| Cal. cb. | Me. viv. | *Rhs. tx.* |
| Carb. v. | *Na. car.* | *Sabadl.* |
| *Chi. of.* | Na. mur. | *Sulphu.* |
| *Ipecac.* | Nux vm. | |

—— " honey=59.
118. Sweet tea: Hep. sl.
119. " " ground: Alumin.
120. Tea: Ast. ru. Cal. sl. Hydras. Pyrus.
—— " ground=119.
—— " sweet=118.
121. Tobacco:

| | | |
|---|---|---|
| Conium. | (Na. car.) | Plumbm. |
| Daph. i. | Nux vm. | Staphs. |
| Eugeni. | (Oxl. ac.) | Therid. |
| Kreoso. | Platin. | Thuj. o. |
| Mancin. | | |

122. " smoking: Carb. a. Glonoi. Ledm. p. Eugeni. Hamame. Lycopo.
123. Unattainable things: Bryoni. Opium.
—— Uncooked flour=50.
—— Undefined=64.
124. Vegetables: Adelhe. *Alumin.* (Chamom.) Alumen. Carb. a. *Mag. cb.*
125. Vinegar. (Compare Acids, 1.) Arnica. Che. ma. *Sepia.* Ars. al. *Hep. sl.*
126. Warm drinks: Angust. Cast. e. Cedron. Me. cor.
127. " food: Angust. Coccul. Cyclam. *Lycopo.* Che. ma. Cu. met. *Fer. mt.* Silicea.

### DESIRE FOR

128. WARM LIQUID FOOD. (Compare SOUPS, 111.) Fer. mt.
129. WATER, COLD:

| | | |
|---|---|---|
| Arnica. | Me. viv. | Rhs. tx. |
| Ars. al. | Na. car. | Ruta. g. |
| Bellad. | Oleand. | Sabadl. |
| Cal. cb. | Phosph. | Sarsap. |
| Copaib. | Platin. | Squill. |
| Ledm. p. | Plumbm. | Thuj. o. |
| Mag. cb. | | |

130. " CRESS: Lepidi.
—— WHEAT BREAD=19.
131. WHISKEY:

| | | |
|---|---|---|
| Acon. n. | Flu. ac. | Pul. pr. |
| *Arnica.* | Hep. sl. | *Seleni.* |
| *Ars. al.* | *Laches.* | *Spigel.* |
| Cal. cb. | LAC. CN. | Staphs. |
| *Carb. a.* | Me. viv. | Sulphu. |
| Chi. of. | Nux vm. | Therid. |
| Cubeba. | Opium. | |

132. WINE:

| | | |
|---|---|---|
| Acon. n. | Cubeba. | Pul. pr. |
| Arg. mt. | Flu. ac. | (Secale.) |
| Bovist. | *Hep. sl.* | Seleni. |
| *Bryoni.* | Ka. bro. | *Sepia.* |
| Cal. cb. | Ka. jod. | *Spigel.* |
| Che. ma. | *Laches.* | Staphs. |
| Chi. of. | Me. viv. | *Sulphu.* |
| *Cicu. v.* | Na. mur. | Therid. |

—— " CLARET=27.

### AVERSION TO

133. ACIDS: Ab. can. *Coccul.* Ignati. Pho. ac. *Sulphu.* *Bellad.* *Fer. mt.* *Nux vm.* *Sabadl.*
134. ALCOHOLIC LIQUORS. (Compare BRANDY, 138, and WINE, 190.) Hippom. Ignati.
—— ANYTHING=155.
135. BANANAS: Elap. c.
136. BEEF: (Me. sul.) (Ptelea.)
137. BEER:

| | | |
|---|---|---|
| Alumin. | Chamom. | Fer. mt. |
| Asafœt. | Chi. of. | Na. mur. |
| Atropi. | Clemat. | *Nux vm.* |
| Bellad. | Coccul. | Phosph. |
| (Bryoni.) | Cro. tg. | Rhs. tx. |
| (Cal. cb.) | Cyclam. | Spigel. |

### AVERSION TO

137. BEER: Spongi. *Stannm.* *Sulphu.*
—— BLACK BREAD=140.
—— BOILED FOOD=160.
—— " MEAT=170.
138. BRANDY. (Compare ALCOHOLIC LIQ., 134.)
*Ignati.* Me. viv. Me. sul. Rhs. tx.
139. BREAD:

| | | |
|---|---|---|
| Agar. m. | Lactuc. | Ol. ani. |
| Chenop. | Lil. ti. | *Phosph.* |
| *Conium.* | *Lycopo.* | *Pho. ac.* |
| *Cyclam.* | Mag. cb. | *Pul. pr.* |
| Elap. c. | Mengan. | *Rhs. tx.* |
| Hippom. | NA. MUR. | *Sepia.* |
| Ignati. | (Na. pho.) | *Sulphu.* |
| Ka. car. | *Nit. ac.* | Tarent. |
| Laches. | *Nux vm.* | |

140. " BROWN: Ka. car. Na. mur.
141. " RYE: *Ka. car.* *Lycopo.* *Nux vm.* Pul. pr *Sulphu.*
142. " WHEAT: Chenop.
143. BREAKFAST: Conium. Lycopo. Mag. sl.
144. BROTH:

| | | |
|---|---|---|
| *Arnica.* | Chamom. | Ka. jod. |
| *Ars. al.* | *Graphi.* | Rhs. tx. |
| Bellad. | | |

—— BROWN BREAD=140.
145. BUTTER:

| | | |
|---|---|---|
| *Ars. al.* | (Mag. cb.) | Petrol. |
| Carb. v. | Mengan. | *Ptel. t.* |
| *Chi. of.* | Me. sul. | Pul. pr. |
| *Cyclam.* | (Na. pho.) | Sangui. |

—— CANDIES=181.
146. CHEESE: *Che. ma.* Oleand.
—— COARSE FOOD=162.
147. CHOCOLATE (or cocoa): Osmium. Tarent.
148. COFFEE:

| | | |
|---|---|---|
| *Bellad.* | Flu. ac. | Osmium. |
| *Bryoni.* | Ka. bic. | (Oxl. ac.) |
| *Cal. cb.* | Ka. nit. | *Phosph.* |
| Carb. v. | Lil. ti. | Physos. |
| *Chamom.* | *Lycopo.* | Rheum. |
| Che. ma. | Me. sul. | Rhs. tx. |
| *Chi. of.* | Me. viv. | *Sabadl.* |
| Coc. ca. | Na. car. | Spigel. |
| *Coff. c.* | *Na. mur.* | *Sul. ac.* |
| Dulcam. | NUX VM. | |

### AVERSION TO

149. COFFEE WITHOUT SUGAR. *Rheum.*
—— COLD FOOD=163.
—— CONFECTIONS=181.
—— COOKED FOOD=161.
150. DINNER: Carb. a. Coc. ca. Ver. al.
151. DRINKS:

| | | |
|---|---|---|
| Agar. m. | (Carb. a.) | Laches. |
| Agns. c. | *Chi. of.* | Me. viv. |
| Aloe s. | Coccul. | Nux vm. |
| Angust. | Coff. c. | Physos. |
| Arnica. | (Conium.) | Ratanh. |
| *Bellad.* | (Fer. mt.) | *Sambucu.* |
| Bufo c. | Hyoscy. | Secale. |
| *Cantha.* | Ignati. | *Stram.* |

—— " ALCOHOLIC=134.
—— " BRANDY=138.
—— " COCOA=147.
—— " COFFEE=148.
152. " HOT: *Fer. mt.*
—— " MALT=137.
—— " TEA=182.
153. " THIRST, WITH. *Lac. ca.* Selin.
—— " WATER=188.
—— " WINE 190.
—— EAT: BREAKFAST=143.
—— " DINNER=150.
—— " FOOD=159.
—— " SUPPER=180.
154. EGGS: Fer. mt.
155. EVERYTHING:

| | | |
|---|---|---|
| Alumen. | (Lycopo.) | Rhodod. |
| Am. mur. | Me. sol. | (Sarsap.) |
| (Bovist.) | Mezeri. | (Sepia.) |
| Capsic. | Nux vm. | Sulphu. |
| Cu. met. | Platin. | Thea. |
| (Gratio.) | Plumbm. | Therid. |
| Hyoscy. | *Pul. pr.* | Thu. o. |
| Ipecac. | Rheum. | |

156. FAT FOOD (or rich food):

| | | |
|---|---|---|
| *Angust.* | Croc. s. | Me. viv. |
| Bellad. | *Cyclam.* | Na. car. |
| *Bryoni.* | Droser. | *Na. mur.* |
| Cal. cb. | Gratio. | PETROL. |
| *Carb. a.* | Hel. ni. | (Phosph.) |
| *Carb. v.* | *Hep. sl.* | PTEL. T. |
| *Colchi.* | *Mengan.* | *Pul. pr.* |

### AVERSION TO

156. Fat food (or rich food): Rheum. Rhs. tx. Sepia. *Sulphu.*
157. Fish: Colchi. *Gratio.* Na. mur. (Sulphu.) *Zn. met.*
—— " herring=168.
158. Flour: Ars. al. *Phosph.* Pho. ac.
159. Food:

| | | |
|---|---|---|
| Ace. ac. | *Colchi.* | Na. slf. |
| Acon. n. | (Conium.) | *Nux vm.* |
| Agar. m. | (Cyclam.) | Oleand. |
| Al. cep. | *Dulcam.* | Ol. ani. |
| Alumen. | Elap. c. | *Opium.* |
| Anacar. | Eu. per. | (Phosph.) |
| *Angust.* | FER. MT. | *Platin.* |
| Ant. cr. | Gambog. | Plumbm. |
| *Ant. ta.* | Graphi. | Prun. s. |
| Arg. mt. | *Gratio.* | *Pul. pr.* |
| Arg. ni. | Guaiac. | Raphar. |
| Arnica. | *Hel. ni.* | Ratanh. |
| Ars. al. | *Ignati.* | (Rheum.) |
| Au. met. | *Ipecac.* | *Rhs. tx.* |
| Baptis. | Ka. bic. | Sabadl. |
| (Bar. ac.) | Ka. car. | Secale. |
| *Bellad.* | Ka. jod. | Sepia. |
| *Bryoni.* | Lactuc. | Silice. |
| Bufo c. | Lauroc. | Squill. |
| (Cal. cb.) | Lycopo. | *Staphs.* |
| *Cantha.* | Mag. cb. | Stront. |
| (Carb. a.) | *Mag. sl.* | Sulphu. |
| Cast. e. | Mangan. | Sul. ac. |
| Chamom. | Me. cor. | Tarent. |
| (Che. ma.) | Me. j. f. | Thea. |
| *Chi. of.* | (Me. sol.) | Tilia. |
| Cimicif. | *Me. viv.* | Ver. al. |
| Cinnab. | Mur. ac. | Wiesba. |
| Coccul. | Na. mur. | Zn. met. |
| (Coc. ca.) | Na. pho. | |

160. " boiled: Che. ma. Lycopo.
161. " cooked:

| | | |
|---|---|---|
| Bellad. | Ignati. | Petrol. |
| Cal. cb. | Laches. | Phosph. |
| Che. ma. | Lycopo. | Silice. |
| Cu. met. | Mag. cb. | Ver. al. |
| Graphi. | Me. viv. | Zn. met. |

162. " coarse: Ab. can.
163. " cold: Ace. ac. Che. ma. Cyclam.
164. " hot: Fer. mt.
—— " salt=178.

### AVERSION TO

165. FOOD, WARM:

| | | |
|---|---|---|
| Bellad. | Laches. | Petrol. |
| Cal. cb. | Lycopo. | Silice. |
| Cu. met. | Mag. cb. | Ver. al. |
| Graphi. | Mag. sl. | Zn. met. |
| Ignati. | Me. viv. | |

166. FRUIT: Bar. cb. Ignati.
—— " BANANAS=135.
—— " PLUMS=174.
167. GARLIC: *Sabadl.*
168. HERRING: Phosph.
—— HOT DRINKS=152.
—— " FOOD=164.
—— LIQUIDS=151.
—— LIQUORS, ALCOHOLIC=134.
—— " MALT=137.
—— MEALS, BREAKFAST=143.
—— " DINNER=150.
—— " SUPPER=180.
—— " FOOD=159.
—— MEAL (flour)=158.
169. MEAT:

| | | |
|---|---|---|
| *Ab. can.* | *Fer. mt.* | Nux vm. |
| Agar. m. | *Graphi.* | Ol. ani. |
| Aloe s. | Hel. ni. | Opium. |
| Alumen. | Hippom. | PETROL. |
| Alumin. | Hydras. | *Platin.* |
| Am. car. | *Ignati.* | *Ptel. t.* |
| Angust. | Ka. car. | Pul. pr. |
| *Arnica.* | Lachna. | *Rhs. tx.* |
| *Ars. al.* | Lactuc. | Ruta. g. |
| Au. met. | *Lycopo.* | *Sabadl.* |
| Bellad. | Mag. cb. | *Sepia.* |
| *Bryoni.* | Mag. sl. | SILICE. |
| *Cal. cb.* | *Me. viv.* | Stront. |
| *Carb. v.* | *Mezeri.* | SULPHU. |
| Caustc. | MUR. AC. | Tarent. |
| Chamom. | Na. car. | Terebi. |
| Chenop. | *Na. mur.* | Zn. met. |
| Coc. ca. | Niccol. | |
| Elap. c. | *Nit. ac.* | |

—— " BEEF=136.
170. " BOILED: *Che. ma.* Nit. ac.
—— " PORK=175.
—— " VEAL=186.

### AVERSION TO

171. MILK:

| | | |
|---|---|---|
| Aethus. | *Carb. v.* | Phosph. |
| Am. car. | *Cina.* | *Pul. pr.* |
| *Ant. ta.* | *Guaiac.* | Rheum. |
| Arnica. | *Ignati.* | *Sepia.* |
| Bellad. | Mag. c. | Silice. |
| *Bryoni.* | *Na. car.* | Stannm. |
| *Cal. cb.* | Nux vm. | *Sulphu.* |

172. " MOTHERS (nursing):

| | | |
|---|---|---|
| Ant. cr. | Me. viv. | Stannm. |
| Cina. | *Silice.* | Stramo. |
| Laches. | | |

—— MOTHERS' MILK=172.
—— NURSING=172.
173. PICKLES: *Ab. can.*
174. PLUMS: Bar. cb.
175. PORK: Angust. *Colchi.* Droser. *Psorni.*
176. POTATOES: Thuj. o.
177. PUDDINGS: Ars. al. Phosph.
—— RICH FOOD=156.
—— RYE BREAD=141.
178. SALT FOOD: Ace. ac. *Carb. v.* *Graphi.* Na. mur. *Seleni.* Silice.
—— SMOKING=184.
—— SNUFFING=185.
—— SOLID FOOD=159.
—— SOURS (sour things)=133.
179. SOUR-KROUT: Hel. ni.
—— SOUPS=144.
—— SPIRITUOUS LIQUORS=134.
—— SUGAR=181.
180. SUPPER: Lycopo. Sulphu.
181. SWEETS:

| | | |
|---|---|---|
| *Ars. al.* | Hippom. | *Phosph.* |
| Bar. cb. | Me. sol. | *Sulphu.* |
| *Causte.* | *Me. viv.* | Zn. met. |
| *Graphi.* | | |

182. TEA: Car. ac. Thea.
183. TOBACCO:

| | | |
|---|---|---|
| Ant. ta. | Carb. a. | Lycopo. |
| Arnica. | Chlora. | Mag. sl. |
| Bovist. | Cimicf. | Mephit. |
| Bromin. | Coccul. | Na. mur. |
| *Cal. cb.* | Conium. | Nux vm. |
| Camphr. | *Ignati.* | Phosph. |
| Cantha. | Laches. | Pul. pr. |

### AVERSION TO

183. TOBACCO: Spigel. Taraxi. Tilia.

184. " SMOKING:

| | | |
|---|---|---|
| Alumin. | IGNATI. | Opium. |
| Asarum. | (Ka. bic.) | (Oxl. ac.) |
| Bromin. | Ka. nit. | Phosph. |
| Bryoni. | Laches. | (Psorin.) |
| Cal. ph. | Lycopo. | *Pul pr.* |
| Carb. a. | Mag. sl. | Sepia. |
| Clemat. | Na. slf. | Spigel. |
| Coff. c. | Niccol. | Tarax. |
| Euphra. | Oleand. | Tellur. |
| Gratio. | | |

185. " SNUFFING: Spigel.

186. VEAL: Phellan. *Zn. met.*

187. VEGETABLES: Bellad. *Hel. ni.* Hydras. *Mag. cb.* Mag. mu. Ruta g.

—— WARM FOOD=165.

188. WATER:

| | | |
|---|---|---|
| *Bellad.* | Chi. of. | (Me. cor.) |
| Bromin. | Coc. ca. | *Na. mur.* |
| *Bryoni.* | Elap. c. | *Nux vm.* |
| *Caladi.* | Hamame. | Oxl. ac. |
| Cantha. | Hel. ni. | *Physos.* |
| Carlsbd. | *Hydrph.* | *Stramo.* |
| Caustc. | Ka. bic. | Thea. |
| (Cedron.) | Lycopo. | |

189. " COLD:

| | | |
|---|---|---|
| Bellad. | Che. ma. | Nux vm. |
| Bryoni. | Chi. of. | *Phella.* |
| *Caladi.* | Hydrph. | *Stram.* |
| Cantha. | Na. mur. | Tabacm. |
| Caustc. | | |

—— WHITE BREAD=142.

190. WINE. (Compare Alcoholic Liq.=134.)

| | | |
|---|---|---|
| Agar. m. | Laches. | Nux ju. |
| Flu. ac. | *Me. sol.* | *Rhs. tx.* |
| Hippom. | *Me. viv.* | *Sabadl.* |
| Ignati. | Na. mur. | Sulphu. |
| Jatrop. | | |

[See Over.]

### PHYSICIANS' SPECIAL RATE CHECKS.

### ADVANTAGES IN THEIR USE:

1. A delicate way to insist upon payment from suspicious persons.
2. *Special* cases can thus be charged at an *advanced* rate.
3. The *poor* can be accommodated with a *reduced* rate.
4. Strangers will be compelled to "stick" until you cure, or convince them that you *can* cure.
5. It discourages the bad habit of "booking" office charges, by offering an inducement for advance payment.

### PRICE, - FIFTY CENTS.

Copyright 1884, by Wm. Jefferson Guernsey, M. D.

Address DAVID HESTON, "Station F," Philadelphia.

[See Over.]

The charges for attendance upon venereal cases should *never* be "*booked*," nor should they be treated at the fee rate for ordinary sickness. By means of these Checks all such patients, (and special cases of any character,) may be required to pay in advance for 10 prescriptions, which may be limited to a given time if desired. Cases of "doubtful honesty" should receive such a prescription. The checks can be used also for all general office practice if thought advisable, but it is the classes above named, particularly the "sinners" that should be compelled to use the Physicians' Special Rate Check. For them there is no better scheme.

For Sale by

J. B. Lippincott & Co., 715 Market St., Phila.
P. Blackiston, Son & Co., 1020 Walnut St., Phila.
Boericke & Tafel, 1011 Arch St., Phila.

PRICE, 50 CENTS
PER BOOK,
OF 25 CHECKS.

[See Over.]

*Sent Post Paid, on receipt of price, by*

DAVID HESTON,

STATION F, PHILA.

Fac-Simile of Check.

PHYSICIANS'

### Special Rate Check.

The coupons attached to this check will be accepted as payment for Office Prescriptions, subject to the following conditions:

1. To be used only by

---------------------------------------------

or a member of his or her family.

2. To be used only for ordinary, non-surgical cases

3. To be presented at each visit to the Office, or regular cash fee paid.

4. Good only for a period of ---------------

5. Attendance to be required only during Office Hours.

